# A Case of Combined Diabetic Ketoacidosis and Hyperosmolar Hyperglycemic State in a Patient With COVID-19

**DOI:** 10.7759/cureus.8965

**Published:** 2020-07-02

**Authors:** Shemitha Rafique, Fahad W Ahmed

**Affiliations:** 1 Diabetes and Endocrinology, Brighton and Sussex University Hospital, Brighton, GBR; 2 Diabetes and Endocrinology, Brighton and Sussex County Hospital, Brighton, GBR

**Keywords:** covid 19, diabetic ketoacidosis, hyperosmolar hyperglycaemic state, hyperglycemic emergency, diabetes mellitus, diabetes, diabetes type 2, type i diabetes mellitus

## Abstract

The novel coronavirus disease 2019 (COVID-19) is caused by the severe acute respiratory syndrome-corona virus-2 (SARS-CoV-2). Diabetes mellitus (DM) is one of the risk factors associated with severe illness in COVID-19 leading to increased hospital admissions and mortality. COVID-19 can precipitate hyperglycemic emergencies like diabetic ketoacidosis (DKA) and hyperosmolar hyperglycemic state (HHS) in patients with DM. We present a case of a patient with COVID-19 admitted to the hospital with combined DKA and HHS. The case highlights the challenge of managing patients with DM suffering from COVID-19.

## Introduction

The pandemic caused by a novel coronavirus named coronavirus disease 2019 (COVID-19) by the World Health Organization has created immense pressure and challenge for all healthcare professionals. As of 15th June 2020, there have been more than 7.9 million confirmed cases with over 434,793 deaths worldwide [1]. Co-morbidities like diabetes mellitus (DM), cardiovascular disease, obesity, and hypertension are associated with severe illness, morbidity, and mortality [2]. In clinical practice, there have been reports of patients with COVID-19 presenting with diabetic ketoacidosis (DKA) and hyperglycemic hyperosmolar state (HHS) in those with previously well-controlled DM [3-6]. However, the evidence is still emerging regarding hyperglycemic emergencies in patients with COVID-19.

## Case presentation

A 79-year-old lady with Alzheimer’s dementia and type 2 diabetes mellitus (T2DM) was found confused and short of breath. She had been coughing for the last few days. Her medications included metformin (500 mg twice a day) and donepezil (10 mg once a day). Prior to admission, her HbA1c was 52 mmol/mol.

She was found to be dehydrated and drowsy, with a Glasgow Coma Scale of 13. She had a respiratory rate of 40/minute and required four liters of oxygen/minute to maintain oxygen saturation 94%. Her heart rate was 110/minute with new atrial fibrillation on the electrocardiogram. There were scattered crackles in the lung bases.

Her initial blood gas showed metabolic acidosis with a pH of 7.19 (7.35-7.45), bicarbonate (HCO3-) of 9.8 mmol/L (22-29 mmol/L), glucose of 41.6 mmol/L, and blood ketones were elevated at 5.1 mmol/L. Other blood results showed an acute kidney injury (AKI) with urea of 17.4 mmol/L (2.76-8.07 mmol/L), creatinine of 193 µmol/L (44-80 µmol/L), and sodium of 152 mmol/L (136-145 mmol/L). The calculated serum osmolality was elevated at 357 mOsm/kg. There was a lymphopenia of 0.9 x 109/L (1.0-3.0 x 109/L), C-reactive protein (CRP) 45 mg/L (<5 mg/L), D-dimer 1.41 ug/mL (0.81-1.45), and ferritin of 1044 mcg/L (13-150).

**Figure 1:**
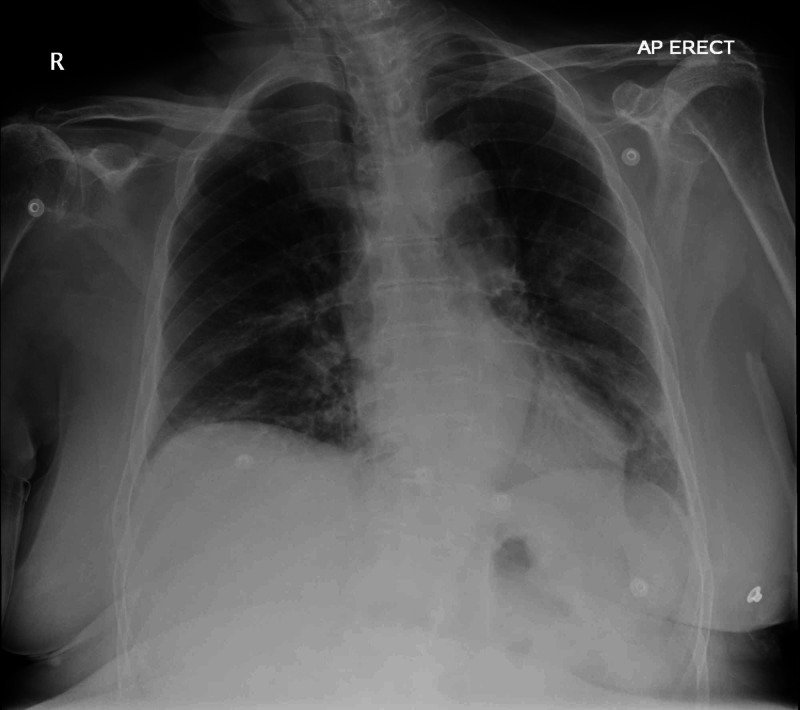
Chest X-ray. Rotated AP view. There are patchy peripheral airspace changes in the left middle, left lower and right lower zones, which are in keeping with COVID-19 infection.

Her chest radiograph showed patchy peripheral airspace changes in the left middle, left lower and right lower zones, which were in keeping with COVID-19 infection (Figure 1). Her nasopharyngeal and throat swab confirmed severe acute respiratory syndrome-corona virus-2 (SARS-CoV-2) infection. 

She was treated with IV levofloxacin and oral doxycycline. In addition, she was treated with IV fluid and insulin infusion protocol (0.05-0.1 units/kg per hour: aiming for a reduction in blood glucose of <5 mmol/L per hour and ketones of at least 0.5 mmol/L per hour). At 24 hours, combined DKA and HHS resolved (blood ketones of 0.1 mmol/L and pH of 7.35). On day two, she was switched to subcutaneous insulin (Humulin I 20 units twice a day). On day three, Humulin I was increased to 30 units twice daily. Bolus novorapid was used if blood glucose was ≥14 mmol/L. Blood ketones and serum electrolytes were closely monitored. 

On day four, she developed hypoglycemia with a capillary blood glucose of 2.9 mmol/L. Her insulin was stopped and 5% dextrose infusion was commenced at a rate of 125 mL/hour. In the following days, she did not need any insulin, and capillary blood glucose was between 7 and 9 mmol/L.

**Figure 2:**
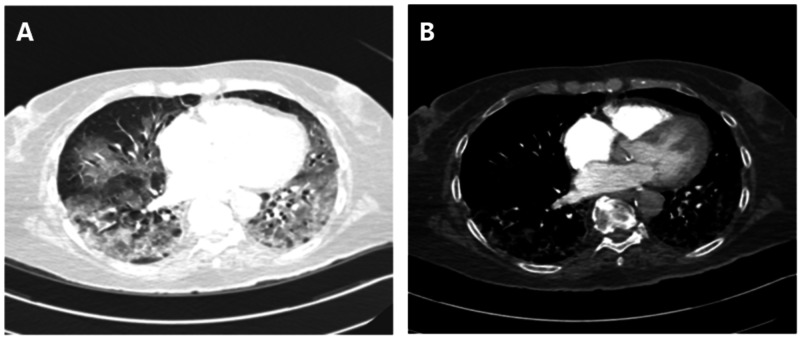
CTPA. A: Lung window demonstrates an appearance in the chest in keeping with classic COVID-19. B: Shows no central PE. CTPA, CT pulmonary angiogram; PE, pulmonary embolism

However, five days into her admission, she became increasingly short of breath with increasing oxygen requirements. Computer tomography angiogram pulmonary (CTPA) was undertaken, which showed extensive bilateral ground-glass change predominantly in the lower lobes, in keeping with COVID-19 infection (Figure 2). There was no evidence of pulmonary embolism. Procalcitonin was 0.25 ng/mL (0-0.5).

Figure 2 shows CT angiogram pulmonary (CTPA) and no central pulmonary embolism (PE). Appearances in the chest are in keeping with classic COVID-19.

Over the next few days, she deteriorated, and CRP rose to 228. Due to her co-morbidities and frailty, she was not a candidate for invasive ventilation. She was, therefore, managed conservatively. On day 15, with family involvement, the patient was transferred to a nursing home. She died five days later.

## Discussion

The case highlights the significant challenge faced by healthcare professionals in managing metabolic abnormalities in patients with DM suffering from severe COVID-19.

Before the presentation, our patient had good glycemic control. Despite this, our patient presented with a hyperglycemic emergency of combined DKA and HSS. In patients with DM and severe COVID-19, it has been proposed that severe hyperglycemia can occur due to severe insulin resistance with beta-cell dysfunction leading to insulin deficiency and/or increased counter-regulatory response [7]. This can result in treatment with large doses of insulin to manage hyperglycemia.

After treatment with IV insulin, our patient needed subcutaneous insulin for two days. Subsequently, the patient developed hypoglycemia. Therefore, subcutaneous insulin was stopped. Blood glucose remained stable without insulin, despite the patient’s overall clinical condition worsening. This could likely be explained by the normalization of insulin resistance or insulin production by the pancreas or both.

Current evidence suggests that people with DM are not at increased risk of developing COVID-19 [8]. However, emerging evidence is clear that people with DM are more likely to be admitted to hospital with severe and life-threatening COVID-19 [2-3]. Furthermore, patients with DM were more likely to have prolonged admission [9]. Besides, DM is one of the most common co-morbidities in patients who have died from COVID-19. Recent data from the United Kingdom demonstrated that 26% of patients who died from COVID-19 had DM [10]. There is an association between HbA1c and body mass index (BMI) and death related to COVID-19 in patients with DM [11]. Our patient had good diabetes control, and based on current evidence still had a higher risk of death with an adjusted hazard ratio of 1.50 [11].

Pathophysiology of SARs-CoV-2 in DM is still under investigation. It is currently hypothesized that COVID-19 may have a diabetogenic effect in addition to the stress-related response of glucose metabolism in severe illness [12]. It is currently proposed that angiotensin converting enzyme 2 (ACE2) may play a role in the pathophysiology of glucose dysregulation in patients with COVID-19 [12]. We now understand that SARS-CoV-2 uses ACE2 as a ligand to bind to its target cells [13]. ACE2 is also expressed on the pancreas as well as other vital organs like lungs and small intestine [14]. SARS-CoV-2 in pancreatic cells can potentially cause direct beta-cell damage [15]. ACE2 expression has been shown to be downregulated once SARS-CoV-2 enters the cell [16]. This could lead to dysregulation of insulin secretion [17]. Another proposed mechanism is an exaggerated response of pro-inflammatory cytokine, interleukin 6 (IL-6) [3]. One of the observational studies has demonstrated that IL-6 levels were significantly higher in patients with DM when compared to patients without DM [18]. A small trial had recently demonstrated a positive outcome with a monoclonal antibody against IL-6. In this study, there were four patients with DM (4 out of 15). All four patients had clinical improvement after the administration of a monoclonal antibody against IL-6, tocilizumab [19].

## Conclusions

Patients with DM may not be at increased risk of contracting COVID-19 but are more likely to develop severe COVID-19 infections leading to increase hospital admissions and mortality. Hyperglycemic emergencies like DKA and HHS appear to be more frequently associated with severe COVID-19. Our case highlights the importance of the dynamic management of glucose metabolism dysfunction. Patients with DM suffering from COVID-19 may require a high dose of insulin to control blood glucose. However, the insulin requirement may reduce drastically, as was the case in our patient, where insulin treatment needed to be withdrawn to prevent recurring hypoglycemia.
